# Academy of Stomatology

**Published:** 1902-07

**Authors:** 

**Affiliations:** Academy of Stomatology


### ACADEMY OF STOMATOLOGY.

A REGULAR meeting of the Academy of Stomatology of Philadelphia was held on the evening of Tuesday, November 26, 1901, at the rooms of the Academy, 1731 Chestnut Street.

A paper was read by Dr. Otto E. Inglis, entitled "Some Peculiar Cases of Dental Resorption."

(For Dr. Inglis's paper, see page 479.)

### DISCUSSION.

*Dr. James Truman.*—I wish to express my satisfaction with the paper, because it covers in the main the facts that nearly all of us are familiar with, while some of them are very interesting owing to their rarity. I was rather disappointed that Dr. Inglis did not give us some experimental facts relating to the causes of this resorption.

The statement that the giant-cells produce it is not satisfactory to my mind. Whether resorption is produced by the action of giant-cells through the elimination of acid secretions, or whether it is due to phagocytic action, is as yet obscure. As I view it, it is something like the separation of necrosed from living bone, and while that has nothing to do with the subject particularly, yet there is a phagocytic action involved in the destruction of the sequestrum. It has never been explained why it is that necrosis will go on in the bone up to a certain point and then cease altogether. I believe the separation is due to a phagocytic activity upon the part of the cells in the wall of circumvallation which produces a resorption of bone at the dividing line similar to that resorption which occurs in deciduous roots, and when that is accomplished then the dead bone separates from the living.

I could wish, in this connection, that some one younger than myself, perhaps Dr. Inglis, would take hold of this question and come to some absolute decision upon it. The detail of cases, while very interesting, does not clear up the subject.

*Dr. J. H. Gaskill.*—Dr. Inglis referred to a case that I reported at Old Point Comfort in 1897, which was rather interesting, because it was, at that time, considered rare. Two teeth were affected in the mouth of the same patient. The dentine of the first tooth resorbed, the lingual enamel crushed in, and the pulp had to be taken out, but when the pink spot indicative of resorption appeared in the second tooth I put the patient under systemic treatment. The pink spot gradually disappeared. I have not seen the patient for several years, but the last time he was in it was not perceptible, while before you could see it from across the room. Since then I have had other cases which have been quite interesting to me and which I think will explain some of the obscure cases of odontalgia. I recall to mind the case of a young lady, who complained of two first bicuspids that had been filled with amalgam and had given a great deal of trouble. I removed the fillings, but could not find any cause, and so I filled them with gold. That did not give any relief. Finally I destroyed the pulps, and for a short time that acted fairly well, but about three years later the teeth had to be extracted. I found three lines below the gum margin an opening in the root which extended into the pulp-canal. The gums were firmly attached at the cervix. There had been resorption and the pulp exposed from without in, and the pain was undoubtedly due to the exposure. I have another patient whom I am watching with a great deal of interest. There is a little resorption of the gum over the first molar at the anterior buccal root. The tooth has no break in its exterior surface, but there is a decided pink spot showing through. These cases throw some light upon obscure cases of odontalgia.

*Dr. A. P. Fellows.*—I have been very much interested in the subject, and I have a couple of specimens to show, corresponding in many respects to those we have just seen. I had a case the other day in the mouth of an individual suffering from pyorrhœa alveolaris. The root end was very much resorbed, seemingly due to inflammation about the root, caused by the deposition of calculus. Dr. Inglis has just stated that such resorptions are probably due to irritation. In this particular case it seemed very probable. The appearance of this resorption area is somewhat peculiar. It seems that secondary dentine has first been formed within the pulp-canal of this tooth to such an extent as almost to obliterate the canal. Following that, the resorption has removed the apical half of the root entirely. The cervical half has had all its normal root dentine resorbed, but the cementum and the secondary dentine remain intact or nearly so. Looked at from the end the root has the appearance of having been bored by a trephine, the central core being left standing *in situ.* I have here another specimen taken from the mouth of a lady fifty years old, who had worn a partial denture for many years. I noticed a small tumor in the vault of the palate, but did not diagnose it correctly owing to the fact that many of the teeth had been extracted. I made a denture, and about a year later the lady complained of irritation about this tumor. I found that the plate was rubbing pretty hard upon it, and upon exploration I found a tooth which I removed. The gum covered the entire tooth, so that the saliva and secretions of the mouth were not in contact with the crown at all. There was absolutely no decay. After removal I found the enamel very badly resorbed. I think that for this particular case it is proved conclusively that it is due to irritation.

*Dr. M. H. Cryer.*—I did not expect to say anything on the subject, but I happened to remember that I have in my pocket a tooth which was removed this afternoon by the aid of the surgical engine. The tooth was not in sight at all. It was the third lower molar, and rested against the distal surface of the second molar. I find the lower cusp apparently somewhat abraded. Now, I am certain that when the distal surface of the second molar is examined, after the wound is cured, it will be found that resorption has taken place, partly owing to the forward pressure or friction that has occurred.

*Dr. William H. Trueman.*—This is a very good paper and on a very interesting subject, but one that is very frequently difficult to discuss. The specimens are also very nicely prepared, being put on cards so that we can understand them; but I feel that justice cannot be done to them in the few minutes examination of them. The subject of resorption is an important one to understand and has many phases. Sometimes it is physiological and beneficial, and at other times it is pathological and mischievous. It has its practical bearing in a case where some careless fellow has killed a pulp and pushed gold through the end of the root of a tooth, and which then lasts from ten to fifteen years. I have never heard it explained how the tooth which remains perfectly comfortable for ten or twelve years could afterwards become uncomfortable and have to be extracted. I extracted a while ago a tooth which had a crown placed upon it. It had been giving considerable trouble and did not respond to remedial treatment at all. I found the root was malformed and a dowel passed through the root about an eighth of an inch. The one who put it in was not a careless fellow, because I did it myself. I did it thirty years ago? No doubt that was a case of resorption, and no doubt many such cases are. I think that one of the cards illustrates it.

*Dr. H. Roberts.*—I would like to know whether Dr. Inglis makes any distinction between resorption of the root of a deciduous tooth as a physiological condition and the resorption of the root of a permanent tooth, which comes from abscess, as a pathological condition.

*Dr. Inglis.*—As to the difference between physiological resorption and pathological resorption, there evidently must be some difference in the primary causes, but not necessarily in the proximate cause of the resorption. Physiological resorption takes place as a physiological process. It is necessary that a tooth should be gotten rid of, and yet it is evident that there is something to a degree pathological in the condition, for so long as the condition of hyperæmia is not set up by some cause these roots are not, as a rule, resorbed. When sufficient but not too great irritation is set up, then the roots are resorbed. We see that in cases where the roots of vital deciduous teeth are not resorbed perhaps for many years after the proper time. I mentioned several cases where cuspids were retained. I mentioned one case where eight deciduous molars are retained in an adult because the bicuspids have not developed and the resorptive action is not yet set up. In the case of chronic abscess the thought was suggested that the resorptions were caused by granulation-tissue produced to repair the loss by suppuration, a condition which could only occur when pus formation is in abeyance, as sometimes occurs in chronic abscesses even to the point of a temporary healing of the fistula. As to the presence of giant-cells in a resorbent organ, Tomes has demonstrated that. Black is also of the opinion that resorption takes place as the result of giant-cells.

*Dr. Schamberg.*—I believe a very interesting example of physiological resorption is the resorption of bone-tissue by pressure from muscles, arteries, and other soft structures which pass over them. I believe it is an acknowledged fact that the facial notch at the angle of the jaw is due to the passage of the blood-vessel over that point, and that while one may, in a measure, inherit this notch, at the same time it is due primarily to the presence there of the artery. There is therefore little doubt in my mind that there is such a thing as physiological resorption. The paper is intensely interesting to me, inasmuch as I am at the present time working upon the reverse condition, exostosis, in an effort to classify the locations of exostosis upon the various portions of the tooth, and to make a study, both macroscopically and microscopically, of its appearance. I am at present endeavoring to stain according to the methods described recently by Dr. Miller. I have not perfected the technic, but hope, at some future date, to present the results of this little study. To this end I would like to ask the members to send me any exostosed teeth they possess which may be worthless to them. I shall be very thankful for the use of them.

*Dr. S. H. Guilford.*—I would say, in regard to this paper, that it strikes me as being a very good one. If we had more of these case papers, they would be of very great value. So often we have peculiar cases in our practice that we lay aside and do not publish. When one prepares a paper on a subject, or an article for one of our magazines, or a book that is coming out, he goes through the literature of the subject and very frequently he does not find what he wants. If all these interesting cases were noted and placed on record in permanent form, they would be of value to those who come after. So that I think Dr. Inglis deserves a great deal of praise for this paper.

*The President.*—Dr. Inglis, will you close the discussion?

*Dr. Inglis.*—Mr. President, the question of resorption of the enamel does not seem to me such a very difficult one, if we may judge from the cases that have been presented in the past and those which we have had this evening. I think that if you will observe these specimens, you will find that not only the enamel is resorbed, but that the bays of resorption extend into the dentine. Dr. Truman prefers to speak of these areas as erosions, and, indeed, I am inclined to believe that the active chemical agent will eventually be shown to be identical in the two conditions. For myself I am inclined to adhere to the nomenclature recently used by Miller in describing a case similar to those which we have observed. It seems more logical to call this condition "resorption," as it occurs in teeth which have never been subjected to oral conditions and under the action of soft tissue irritated to the point corresponding to proliferative activity and commonly called granulation-tissue. The resorbed areas are rough, a condition exactly corresponding to areas produced by continued trickling of acid, such as dilute acid sodium phosphate, over the crowns of extracted teeth. The bays of excavation in the crown correspond to the bays produced in resorbed roots.

In the erosion of enamel and dentine in the mouth there is added to the action of the acid from the labial glands a constant friction of the lips, brush, or food which probably removes the soluble salts resulting from the reaction between the acid and tooth-tissue.

In resorption the salts are also removed, but, lacking friction, the areas remain rough. Perhaps Dr. Cryer's case just mentioned may be an example in which the solvent plus friction has produced a true subtegumental erosion.

INCIDENTS OF PRACTICE.

*Dr. Walter H. Neall.*—Under the order of incidents of practice I would like to report a case of quinine poisoning. When a patient of mine takes quinine he has a high fever and peeling of the skin from his hands and feet. Even his finger- and toe-nails are affected.

I wrote him a letter asking many questions as to the details of his case, and he very kindly replied as follows:

"1. Born in Philadelphia, Pa., December 12, 1854.

"2. First noticed the action of quinine upon me when about twenty-two years of age, at which time my physician informed me that it was 'German measles.'

"3. Every time I caught a cold and was given quinine, among other medicines, I had the 'German measles.'

"4. My physician at one time prescribed a tonic containing quinine, iron, and strychnine; I had another attack, and it occurred to me that the quinine was the cause of the rash and its attending discomforts, so when I was in good condition and free from all colds, etc., I took a two-grain pill of quinine, and that proved conclusively what was giving me so many attacks of 'German measles.'

"5. About two hours after taking even one-half grain I have a high fever, small lumps appear on my arms and chest about the size of a small pea, and I become red as though I had scarlet fever or measles. The effort of breathing is very painful, and I am obliged to have my chest from above the collar bone almost to the umbilicus covered with a hot mustard-plaster. During this time it feels as if some thick substance were being forced through my flesh. This lasts about three hours, after which I can generally sleep.

"6. The skin of my hands and feet can invariably be pulled off two weeks after taking the first dose of quinine.

"7. The finger- and toe-nails seem to be brittle, and as they grow out there is quite a ridge, which is probably formed by a new nail joining the old one.

"8. About three hours after the rash appears it disappears, and I have no further trouble, no matter how long (say a month) I continue taking the quinine. After I have pulled the skin off my hands, which takes only a few minutes when it is ready to come off, I am as good as new, the skin being tender for a few days.

"9. My tongue becomes heavily coated the day after taking quinine. This coating gradually disappears. I have noticed no indications in regard to my liver.

"10. My eyes and ears are affected as though I had a heavy cold.

"11. To me the effect of this drug seems to stop all growth for the time being. I have noticed that my hair also seems to have stopped growing, and does not require trimming for say a month or six weeks. My daughter, who is now over twenty-one years of age, is not affected in any way. My son, who is in his ninth year, has the same experience that I do. I cannot say in regard to my parents; I have never heard through their brothers or sisters that they were affected in any way."

This gentleman appeared in my office within the last two weeks suffering from this quinine poisoning. He had taken certain advertised powders concerning which he had questioned his physician, but which the latter carelessly permitted him to take. A brother practitioner and I examined him. He showed how he could remove his skin with his fingers. He said he could do the same with his feet. He appeared like a person with a very bad case of sunburn without the redness usually attendant. After he stops the quinine his skin will still peel, and the new skin forming will take on the same affection if he renews the quinine treatment. I have not known before anything of this kind, though I know that in some cases the flesh will puff up and there will be great itching. I thought the case would interest you.

Adjourned.

Otto E. Inglis,
*Editor Academy of Stomatology.*

